# Direct Comparison Between Dynamic Conformal Arcs and Volumetric Modulated Arcs in Stereotactic Radiosurgery Planning Using the High-Definition Dynamic Radiosurgery Platform for Intact Brain Metastases

**DOI:** 10.7759/cureus.92983

**Published:** 2025-09-22

**Authors:** Kazuhiro Ohtakara, Kojiro Suzuki

**Affiliations:** 1 Department of Radiation Oncology, Kainan Hospital Aichi Prefectural Welfare Federation of Agricultural Cooperatives, Yatomi, JPN; 2 Department of Radiology, Aichi Medical University, Nagakute, JPN

**Keywords:** agility mlc, brain metastases, dynamic conformal arc therapy (dcat), high-definition dynamic radiosurgery (hdrs), intelligent beam, monaco tps, multileaf collimator (mlc), stereotactic radiosurgery (srs), virtual 1 mm leaves, volumetric-modulated arc therapy (vmat)

## Abstract

Purpose

This planning study aimed to directly compare the quality and efficiency of treatment planning between dynamic conformal arcs (DCA) and volumetric modulated arcs (VMA) in stereotactic radiosurgery (SRS) using the high-definition dynamic radiosurgery (HDRS) platform for unresected solitary brain metastases (BMs).

Materials and methods

All 20 lesions used in a previous study comparing multiple methods for optimizing DCA with the HDRS were reincluded in this study. The main constituents of the HDRS included the 5 mm leaf-width multileaf collimator (MLC) Agility^®^ (Elekta AB, Stockholm, Sweden) and the planning system Monaco^®^ (Elekta AB). Two SRS plans were created for each gross tumor volume (GTV) with DCA and VMA. In both optimizations, the dose conformity and the steepness of the dose gradient outside the GTV boundary were prioritized using the unified methods, with few exceptions, which were currently deemed optimal based on previous studies. After the optimization, each GTV coverage by the same prescription dose was rescaled according to D_V-0.01 cc_, a minimum dose to the GTV minus 0.01 cc. The total calculation time (tCT), the reproducibility of the intended marginal dose, and the quality of dose distribution were compared.

Results

The tCT and the reproducibility of the GTV marginal dose were significantly shorter and higher, respectively, while the total monitor units (MU) per fraction were significantly higher in the VMA, compared with the DCA. The dose distributions of VMA were significantly superior to those of DCA in terms of the GTV dose conformity, the appropriateness of dose attenuation margin, and the steepness and concentric lamellarity of dose gradients outside and inside the GTV boundary. The dose attenuation margin was too steep for some small lesions in both the DCA and the VMA.

Conclusions

Compared to DCA, VMA is less efficient in irradiation; however, the quality and efficiency of treatment planning are significantly superior. VMA was positioned as the top-priority technique for SRS using the HDRS for unresected BMs. However, sufficient consideration must be given to the extremely steep dose attenuation margin and internal dose increase at the GTV boundary, depending on the case.

## Introduction

Stereotactic radiosurgery (SRS) is an essential treatment for brain metastases (BMs) owing to its effectiveness and minimal invasiveness [1,2]. Image-guided frameless, highly accurate fractionated irradiation and inverse planning are recently available, greatly expanding the scope of application for the size and number of lesions [2,3]. SRS can also be performed using a variety of devices and irradiation techniques, although there are now significant differences between devices and facilities in terms of dose distribution, applicable lesion number, and treatment time [1-3]. Even with the same device, differences in planning and dose prescription can have a significant impact on treatment outcomes. The criteria for determining superior treatment include a high induction rate of local remission accompanied by earlier lesion shrinkage and reduction in the surrounding edema and whether radiation injury, if any, is limited to minor imaging changes [4,5]. To maximize the efficacy and safety of SRS, it is essential to determine how to create the optimal dose distribution physically achievable with each equipment.

General-purpose linacs have enabled SRS owing to advances in multileaf collimators (MLCs), six-degree-of-freedom image guidance, and planning systems. The high-definition dynamic radiosurgery (HDRS) platform focuses on the constituents required for frameless SRS using a high-precision irradiation-compatible linac provided by Elekta AB (Stockholm, Sweden) [6-8]. The main constituents for creating the dose distribution include the all 5 mm leaf-width, 160-leaf collimator Agility^®^ (Elekta AB) and the Monaco^®^ treatment planning system (TPS) (Elekta AB) [9,10]. The HDRS can provide both dynamic conformal arcs (DCA) and volumetric modulated arcs (VMA) as SRS techniques [11-13].

DCA has been the standard technique since SRS was first performed using an MLC [14]. The DCA using the HDRS has unique features such as highly accurate dose calculation with the X-ray voxel Monte Carlo (XVMC) algorithm, variable gantry rotation speed, variable dose rate, and inverse planning with segment shape optimization (SSO) [11-13]. Inverse planning with the SSO can be optimized using a combination of multiple physical and/or biological cost functions similar to those used in VMA [11,15,16]. In addition, the SSO enables leaf adaptations to a target volume (TV) boundary with anisotropic leaf margins in which some leaf edges extend medially beyond the TV boundary, i.e., minus leaf margins [15].

The HDRS also allows a high degree of freedom in setting the number of arcs, their arrangement, rotation range, and collimator angle, and it is convenient to create a template for the settings [6-10]. Typically, high dose concentration is achieved by arranging one coplanar arc (CA) and two non-coplanar arcs (NCA) on a trajectory that trisects the cranial hemisphere [17]. Furthermore, by combining different collimator angles, dose distribution optimization can be achieved with beamlet sizes of substantially less than 5 mm [15,17,18]. Among the leaves involved in forming the irradiation field of each segment, the outermost leaves in the leaf traveling direction function with <5 mm variable widths owing to dynamic shielding by diaphragms (jaws), referred to as the virtual 1 mm leaves [15,18,19]. The positions of the leaf tips and the diaphragm ends in each segment are controlled in 0.1 mm increments. Other platforms allow for 90-180 control points, spaced 2°-4° apart, in one 360°-rotation arc, while VMA with the HDRS allows for up to 1,024 control points per arc in increments of <1°, referred to as the intelligent beam. The allocation of monitor unit (MU) values to each arc is optimized individually and unevenly in both DCA and VMA [15]. Although it is often assumed that a 5 mm leaf-width MLC is unsuitable for SRS of small intracranial lesions, the HDRS with the 5 mm leaf-width Agility^®^ can provide a dose distribution sufficient for SRS of small and/or irregular lesions if certain conditions are met in the optimization [20,21].

The authors have pursued methods for creating the most suitable dose distributions for SRS as simply as possible by taking full advantage of the characteristics of the HDRS for VMA and DCA, respectively [15,16]. However, the relative merits of DCA versus VMA specific to the HDRS in terms of the quality and efficiency of treatment planning and delivery are unclear in SRS for BMs. To the best of our knowledge, DCA and VMA using the HDRS have not been directly compared as SRS for BMs [22,23].

This planning study, therefore, aimed to directly compare the quality and efficiency of treatment planning between DCA and VMA in SRS using the HDRS for unresected single BMs. Specifically, the total calculation time (tCT), the reproducibility of an intended marginal dose, the total MU per fraction, and the quality of dose distribution were compared.

## Materials and methods

This study was conducted as part of a comprehensive study approved by the Clinical Research Review Board of Kainan Hospital Aichi Prefectural Welfare Federation of Agricultural Cooperatives (number: 20240830-01). In this study, all 20 lesions from 19 cases harboring BMs, used in the aforementioned previous study on DCA optimization, were intentionally included again [15]. This study corresponds to part 2 of the previous study [15], to facilitate comparison of the quality and efficiency of treatment plans, including dose distributions. The gross tumor volume (GTV) statistics were as follows: minimum 0.33 cc, maximum 48.09 cc, median 7.05 cc, and interquartile range (IQR) 2.08-26.34 cc.

The linac system was Infinity^®^ (Elekta AB) with a flattening filter-free 6 MV X-ray beam. Two SRS plans were created for each GTV: DCA and VMA. The irradiation center was set at the center of each GTV and was consistent for both plans. The arc arrangements for each GTV were set differently for DCA and VMA according to the methods as described previously, with the rotation range of 120° for DCA and a combination of 180° and 360° for VMA [15,16]. The collimator angles were uniformly set to 0°, 45°, and 90° for the CA, left NCA, and right NCA, respectively [16]. All the increment (Inc) parameters were standardized to 20° for each arc [16].

In both DCA and VMA optimizations, conformity of the prescription isodose surface (IDS) to the GTV boundary and the steepness of the dose falloff outside the boundary were given top priority, without any dose constraints within the GTV [16,18]. The optimization methods for DCA and VMA that were applied based on our previous studies are shown in Table 1, which shows the majority of the commonalities and minor differences [15,16].

**Table 1:** The unified optimization settings with common and different parts for dynamic conformal arcs (DCA) and volumetric modulated arcs (VMA). *The minimum volume is the coverage value of each gross tumor volume (GTV) with the prescription dose. IMRT: intensity-modulated radiotherapy; RMS: root mean square; Max: maximum; Min: minimum.

Item	Setting details		
IMRT constraints (Pareto)	Structure	Cost function	Parameter settings
	GTV	Target penalty	Prescription (Gy): 43.000
			Minimum volume (%): 97.00-99.98*
	Patient (body contour)	Conformality	Relative isoconstraint: 0.01
			Margin around target: 8 cm
			Multicriterial +
		Quadratic overdose	Maximum dose (Gy): 43.000
			RMS dose excess (Gy): 0.020
			Multicriterial +
			Shrink structures (cm): GTV 0.20
IMRT prescription parameters	Irradiation technique	DCA	VMA
	Beamlet width (cm)	0.20	0.30
	Target margin	Very tight (0-1 mm)	Normal (8 mm)
	Avoidance margin	Very tight (0-1 mm)	Normal (8 mm)
Sequencing parameters	Segment shape optimization: +		
	High-precision leaf positions: 20		
	Irradiation technique	DCA	VMA
	Differences in settings	Constant dose rate (-)	Max number of arcs: 1
			Max. # of control points per arc: 1,024
			Min. segment width (cm): 0.50
			Fluence smoothing: medium
Calculation properties	Grid spacing (cm): 0.10		
	Calculation dose deposition to: medium		
	Statistical uncertainty (%): 1.00 per calculation		

The standard dose prescription with 43.000 Gy in five fractions was the GTV D_V-0.01 cc_, the minimum dose to the GTV minus 0.01 cc (D_>95%_) [24]. The dose prescription methods were adopted to improve the flaws of prescribing to D_≤98%_ of the GTV or planning TV (PTV) with an isotropic margin [24]. After optimization, each GTV coverage with the prescription dose was rescaled according to the specified coverage value [16]. The tCT was recorded from the optimization console to compare the efficiency of planning [16]. The reproducibility of the GTV marginal dose was determined by the small difference between the rescaling ratio and the reference value 1 as described previously [16].

To compare the dose distributions, dosimetric parameters more relevant to treatment outcomes were adopted rather than common evaluation metrics such as D_98%_, D_50%_, D_2%_, and dose conformity, homogeneity, and gradient indices [16,25,26]. Table 2 summarizes the plan evaluation metrics, their definitions, and how to interpret them.

**Table 2:** Dosimetric parameters used for comparison of dose distributions. *The minimum dose to 0.01 cc (D_<5%_), receiving a near-maximum dose, of a gross tumor volume (GTV). **This metric can also be interpreted as follows: the higher the value, the steeper the dose increase inside the GTV boundary, although the higher value may occur due to over-coverage of the GTV by the prescription isodose. D_V-0.01 cc_: minimum dose to target volume (TV) minus 0.01 cc; IDS: isodose surface; PD: prescription dose; PIV: prescription isodose volume; IIDV: irradiated isodose volume; D_eIIV_: minimum dose to the IIDV equivalent to a TV.

Evaluation items	Metrics	Definitions	Interpretation
GTV dose inhomogeneity	GTV D_V-0.01 cc_ % IDS (%)	PD (%) relative to D_0.01 cc_* (100%)	The lower the value, the more heterogeneous
GTV dose conformity	PIV spillage (cc)	PIV minus GTV	The smaller the value, the better the conformity
	GTV D_eIIV_ (%)	Minimum dose (%) to IIDV equivalent to GTV, relative to PD (100%)	The closer to 100%, the better the conformity**
	GTV D_eIIV_ coverage (%)	GTV coverage (%) by D_eIIV_	The higher the value, the better the conformity
Appropriateness of dose attenuation margin	GTV + 2 mm D_eIIV_ (%)	Minimum dose (%) to IIDV equivalent to GTV + 2 mm, relative to PD (100%)	The lower the value, the steeper the dose decrease (gradient) outside the GTV boundary
Concentric lamellarity of dose attenuation margin	GTV + 2 mm D_eIIV_ coverage (%)	GTV + 2 mm coverage (%) by D_eIIV_	The higher the value, the better the concentric layering of the dose gradient outside the GTV boundary
Steepness of dose gradient outside the GTV	75% or 50% PIV spillage (cc)	IIDV of 75% or 50% of PD minus GTV	The smaller the value, the steeper the dose gradient outside the GTV boundary
Steepness of dose increase inside the GTV boundary	GTV – 2 or 4 mm D_eIIV_ (%)	Minimum dose (%) to IIDV equivalent to GTV – 2 or 4 mm, relative to PD (100%)	The higher the value, the steeper the dose increase inside the GTV boundary
Concentric lamellarity of dose increase inside the GTV boundary	GTV – 2 or 4 mm D_eIIV_ coverage (%)	GTV – 2 or 4 mm coverage (%) by D_eIIV_	The higher the value, the better the concentric layering of the dose gradient inside the GTV boundary

The GTV-2 mm and GTV-4 mm were created only for GTVs of ≥1.26 cc (17 lesions) and ≥1.71 cc (16 lesions), respectively [24]. Statistical analyses were based on paired non-parametric tests and were performed using the BellCurve for Excel^®^ (version 4.05; Social Survey Research Information Co., Ltd., Tokyo, Japan). The distributions of numerical variables were shown as box-and-whisker plots (BWPs), in which the whiskers displayed the nearest values ≤ 1.5 times the IQR, and the cross marks beyond the lines denoted the outliers > 1.5 times the IQR. Two numerical variables were compared using the Wilcoxon signed-rank test (WSRT). Statistical significance was considered at p < 0.05 and expressed on a three-level scale: p < 0.05 (*), p < 0.01 (**), and p < 0.001 (***). Significant p-values were marked in blue in the figures.

## Results

**Figure 1:**
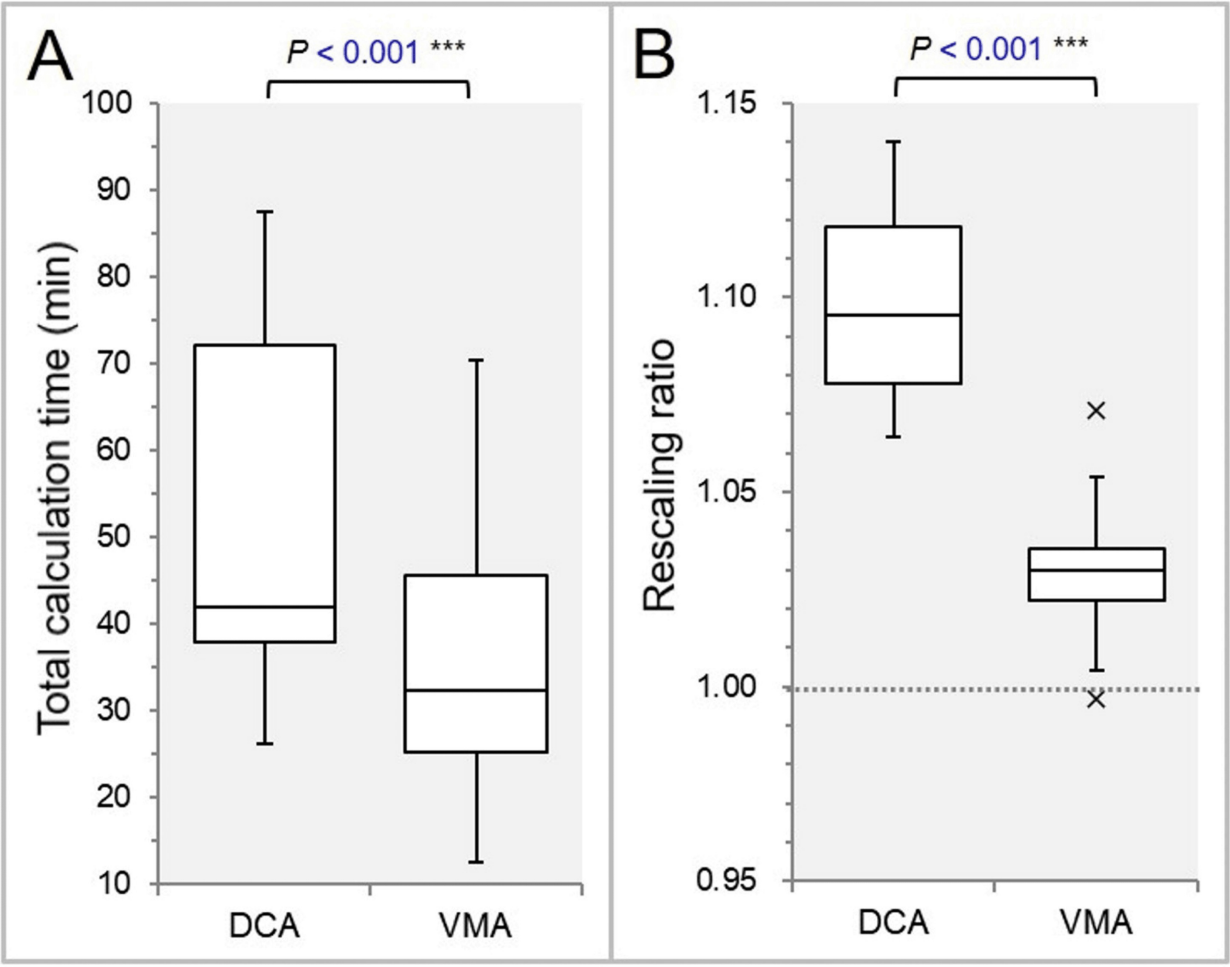
Comparisons of the total calculation time and the rescaling ratio. The box-and-whisker plots (BWPs) show the total calculation times (A) and the rescaling ratios (B). The dotted line in (B) indicates the 1.000 value, meaning no need for rescaling. The results of the Wilcoxon signed-rank test (WSRT) are attached (A, B). DCA: dynamic conformal arcs; VMA: volumetric modulated arcs.

The tCT was significantly shorter, and the rescaling ratio was significantly smaller in the VMA plans (Figures 1A, 1B).

**Figure 2:**
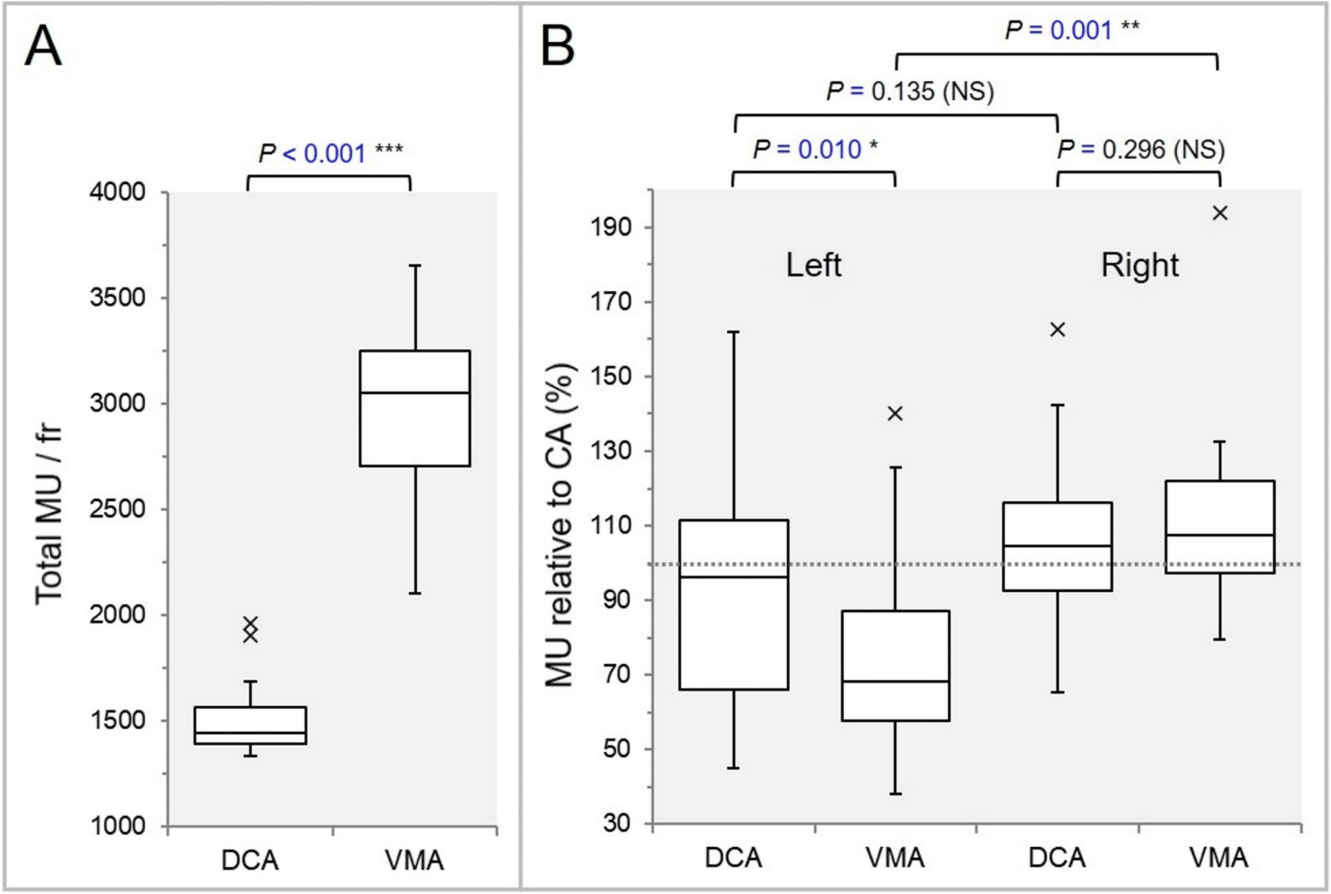
Comparisons of the total monitor units (MU) per fraction and the MU values of each non-coplanar arc relative to those of the coplanar arc. The BWPs show the total MU per fraction (A) and each MU (%) of the two non-coplanar arcs (NCA), relative to that of the coplanar arc (CA, 100%) (B). The dotted line in (B) displays the value (100%) equivalent to that of the CA. The “left” and the “right” show the NCA on the left and right sides of the head, respectively. The results of WSRT are attached (A, B). NS: not significant; BWP: box-and-whisker plot; WSRT: Wilcoxon signed-rank test; DCA: dynamic conformal arcs; VMA: volumetric modulated arcs.

The difference between the rescaling ratio and the reference value of 1 was significantly smaller in the VMA (Figure 1B). The total MU per fraction was significantly higher in the VMA (Figure 2A).

**Figure 3:**
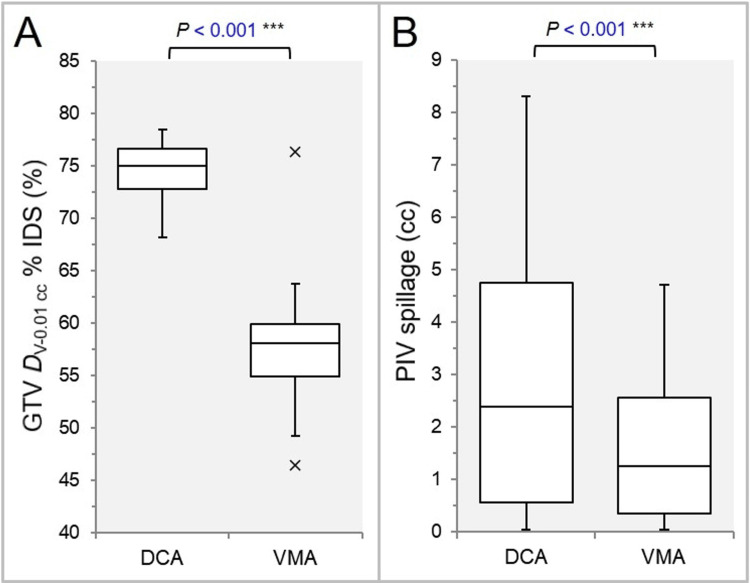
Comparisons of the GTV dose inhomogeneity and dose conformity. The BWPs in (A) show the prescription dose (%) relative to the near-maximum dose (100%). The BWPs in (B) display the prescription isodose volume (PIV) spillage outside the gross tumor volume (GTV). The results of WSRT are attached (A, B). D_V-0.01 cc_: minimum dose to target volume (TV) minus 0.01 cc; IDS: isodose surface; DCA: dynamic conformal arcs; VMA: volumetric modulated arcs; BWPs: box-and-whisker plots; WSRT: Wilcoxon signed-rank test.

The MU ratios of the NCA varied substantially with the ranges from 45.0% to 162.5% and from 38.2% to 193.9% in the DCA and the VMA, respectively (Figure 2B). The MU ratios of the left-sided NCA were significantly smaller in the VMA (Figure 2B). The GTV dose inhomogeneity was significantly greater, and the prescription isodose volume (PIV) spillage was significantly smaller in the VMA (Figures 3A, 3B).

**Figure 4:**
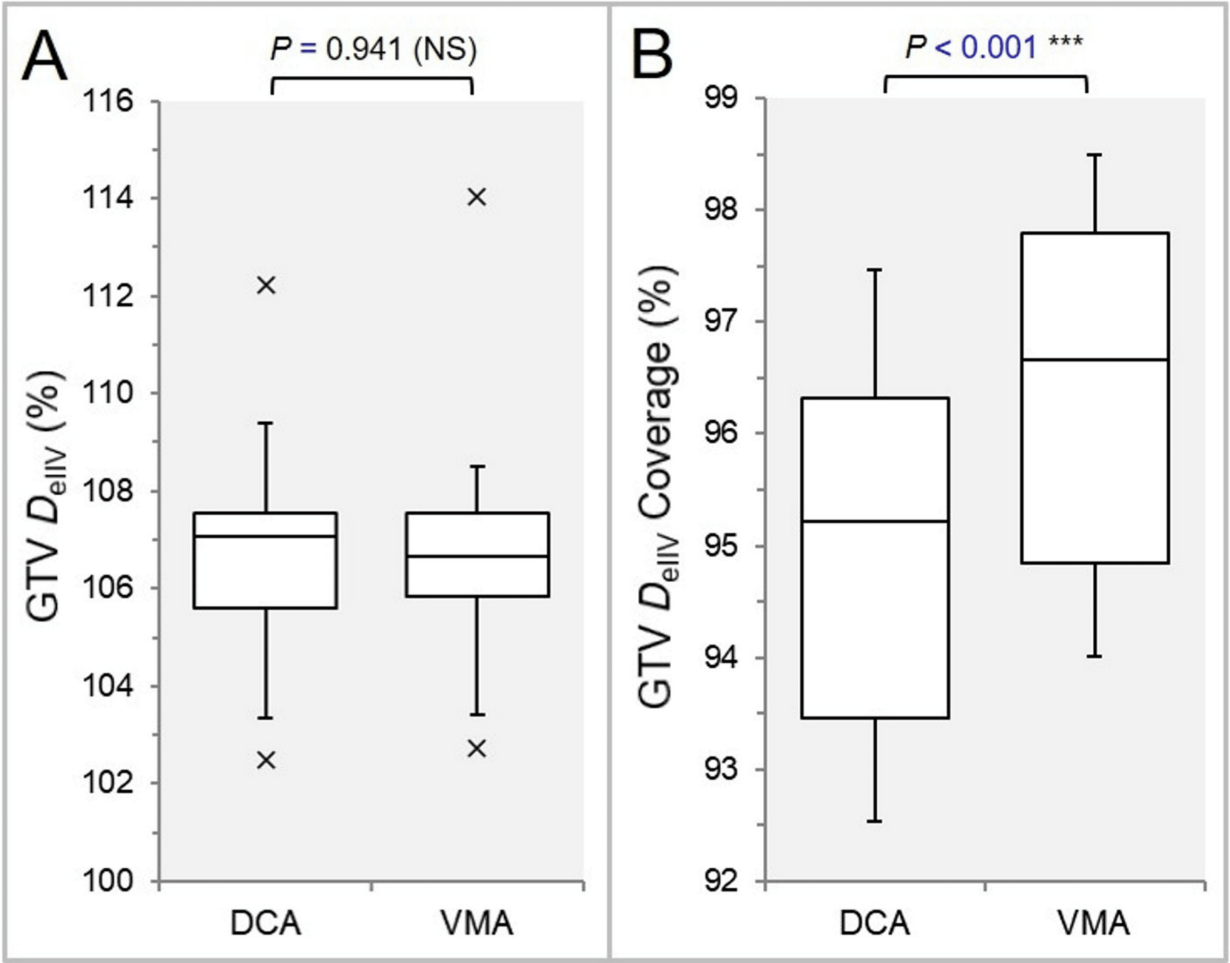
Comparisons of the GTV dose conformity. The BWPs in (A) show the GTV D_eIIV_, minimum dose to the irradiated isodose volume (IIDV) equivalent to the GTV, relative to the prescription dose (100%). The BWPs in (B) display the GTV coverage value (%) by the D_eIIV_. The results of WSRT are attached (A, B). NS: not significant; GTV: gross tumor volume; BWPs: box-and-whisker plots; WSRT: Wilcoxon signed-rank test; DCA: dynamic conformal arcs; VMA: volumetric modulated arcs.

The GTV coverage by the D_eIIV_, the minimum dose to the irradiated isodose volume equivalent to the GTV, was significantly higher in the VMA, although there was no significant difference in the GTV D_eIIV_ (Figures 4A, 4B).

**Figure 5:**
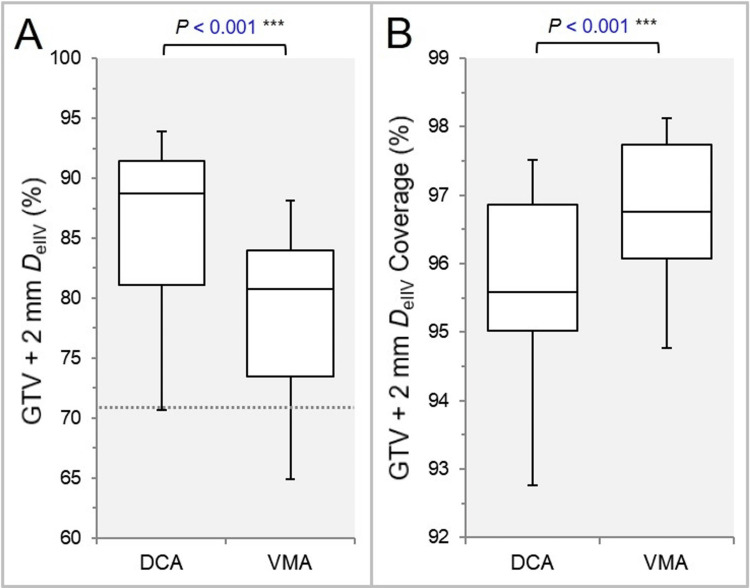
Comparisons of the appropriateness of dose attenuation margin outside the GTV. The BWPs in (A) show the D_eIIV_ (%) of the GTV + 2 mm, relative to the prescription dose (100%). The dotted line in (A) displays the 71.2% value below which the dose falloff outside the GTV boundary becomes too steep. The BWPs in (B) show the GTV + 2 mm coverage value (%) by the D_eIIV_. The results of WSRT are attached (A, B). GTV: gross tumor volume; BWPs: box-and-whisker plots; WSRT: Wilcoxon signed-rank test; DCA: dynamic conformal arcs; VMA: volumetric modulated arcs.

The D_eIIV_ of the GTV + 2 mm was significantly lower, and the coverage value of the GTV + 2 mm by the D_eIIV_ was significantly higher in the VMA (Figures 5A, 5B).

**Figure 6:**
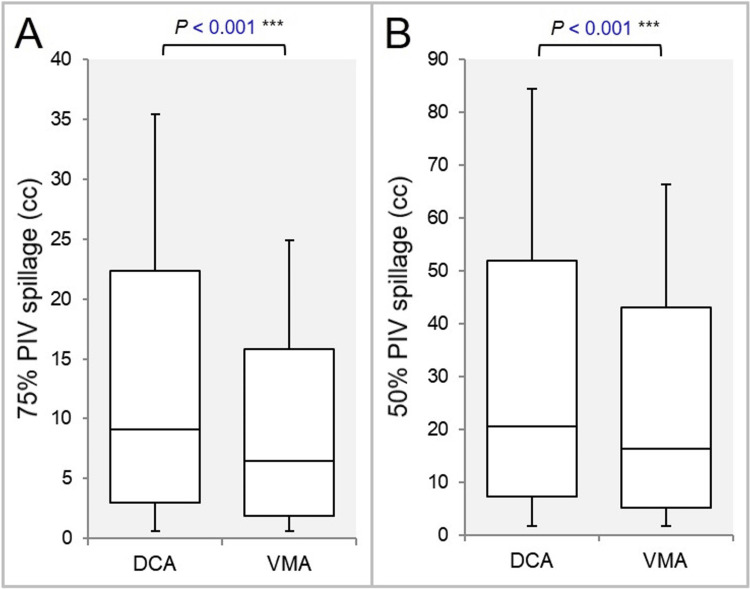
Comparisons of dose gradients outside the GTV boundary. The BWPs show the 75% (A) and 50% (B) PIV spillage outside the GTV. The results of WSRT are attached (A, B). GTV: gross tumor volume; BWPs: box-and-whisker plots; WSRT: Wilcoxon signed-rank test; DCA: dynamic conformal arcs; VMA: volumetric modulated arcs; PIV: prescription isodose volume.

The coverage value was less than 71.2% in one DCA plan (GTV 0.33 cc) and three VMA plans (GTV 0.50, 0.72, and 1.71 cc) (Figure 5A) [25]. The 75% and 50% PIV spillage volumes were significantly smaller in the VMA (Figures 6A, 6B).

**Figure 7:**
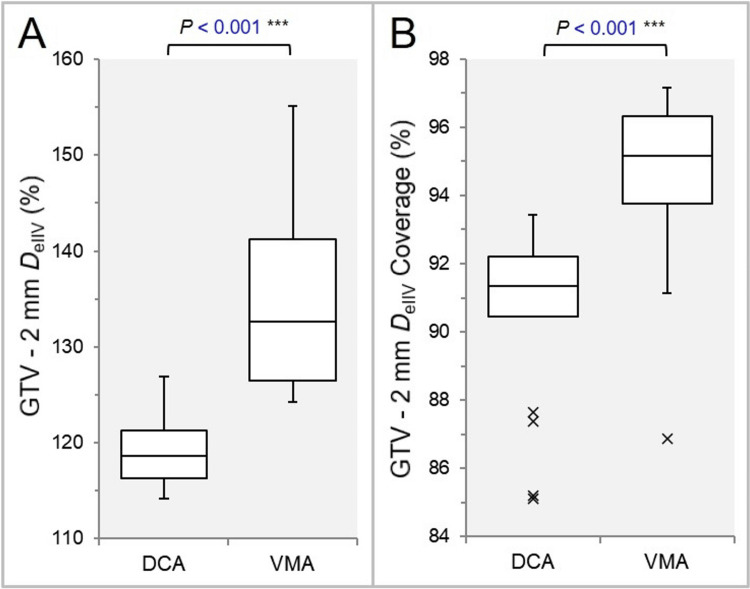
Comparisons of the steepness and the concentric lamellarity of dose increase at 2 mm inside the GTV boundary. The BWPs show the D_eIIV_ (%) of the GTV - 2 mm, relative to the prescription dose (100%) (A) and the coverage value (%) of the GTV – 2 mm by the D_eIIV_ (B). The results of WSRT are attached (A, B). GTV: gross tumor volume; BWPs: box-and-whisker plots; WSRT: Wilcoxon signed-rank test; DCA: dynamic conformal arcs; VMA: volumetric modulated arcs.

The D_eIIV_ of the GTV - 2 mm was significantly higher, and the coverage value of the GTV - 2 mm by the D_eIIV_ was significantly higher in the VMA (Figures 7A, 7B).

**Figure 8:**
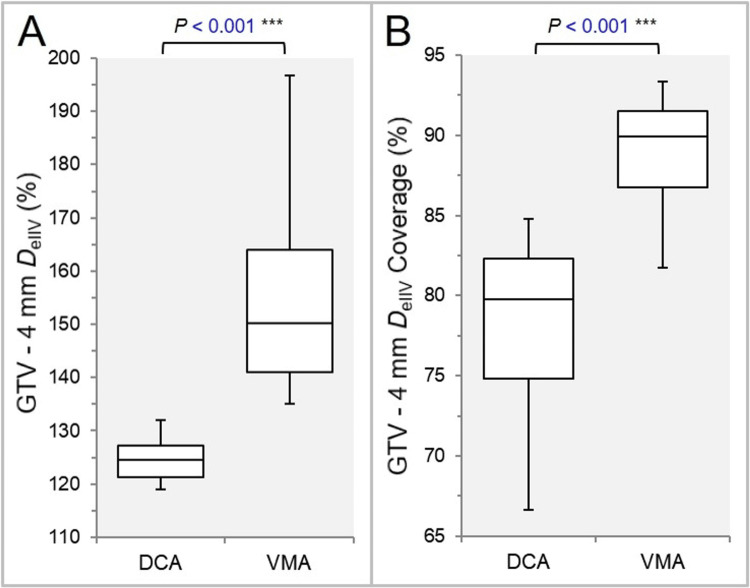
Comparisons of the steepness and the concentric lamellarity of dose increase at 4 mm inside the GTV boundary. The BWPs show the D_eIIV_ (%) of the GTV - 4 mm, relative to the prescription dose (100%) (A) and the coverage value (%) of the GTV – 4 mm by the D_eIIV_ (B). The results of WSRT are attached (A, B). GTV: gross tumor volume; BWPs: box-and-whisker plots; WSRT: Wilcoxon signed-rank test; DCA: dynamic conformal arcs; VMA: volumetric modulated arcs.

The D_eIIV_ of the GTV - 4 mm was significantly higher, and the coverage value of the GTV - 4 mm by the D_eIIV_ was significantly higher in the VMA (Figures 8A, 8B).

**Figure 9:**
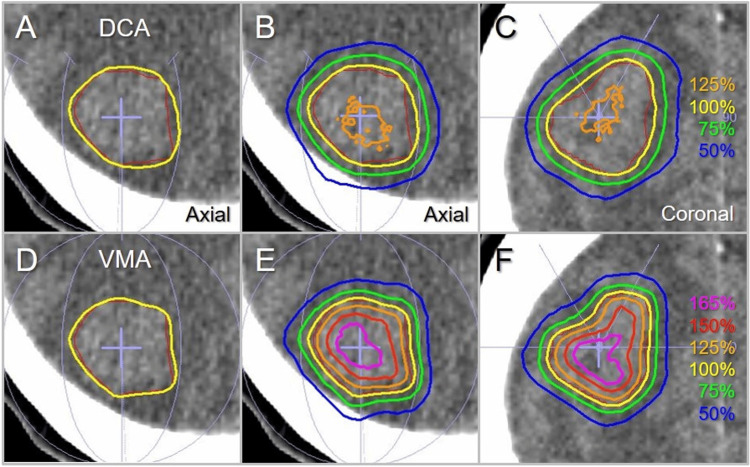
Comparison of the dose distributions for GTV of 9.54 cc. The images show computed tomographic images (axial views: A, B, D, E; coronal views: C, F) of the case harboring a brain metastasis in the right parietal lobe (A-F), onto which the GTV outline in red, arc arrangements in light purple, and the representative isodose lines for DCA (A-C) and VMA (D-F) are superimposed. The isodose lines are shown as the relative values to the prescription dose (100%) in yellow. GTV: gross tumor volume; DCA: dynamic conformal arcs; VMA: volumetric modulated arcs.

Figure 9 shows the representative dose distributions for the GTV of 9.54 cc with irregular shape, which was previously described in a case report and was also used in a previous planning study to compare dose distributions [17,27].

**Figure 10:**
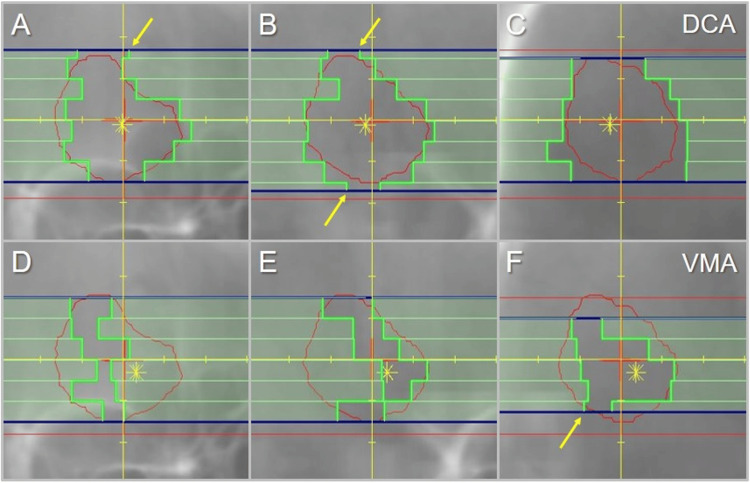
Comparison of the beam’s eye views of the coplanar arcs for the GTV of 9.54 cc. The images show the beam’s eye views (A-F) of the representative three segments of the coplanar arcs for DCA (A-C) and VMA (D-F). Arrows (A, B, F) display the <5 mm variable widths of some of the outermost leaves involved in the segment formation in the leaf movement direction, through dynamic shielding by the diaphragms in dark blue. The asterisks show each highest dose area. DCA: dynamic conformal arcs; VMA: volumetric modulated arcs; GTV: gross tumor volume.

The dose conformity of the prescription IDS to the GTV boundary and the steepness and the concentric lamellarity of the dose gradients both outside and inside the boundary were clearly superior in the VMA (Figure 9). A comparison of the representative beam segments of the coplanar arcs is shown in Figure 10.

The VMA beam segments formed irregular partial apertures within the GTV contour, compared to those close to the GTV boundary in the DCA.

## Discussion

Compared to DCA, VMA was overwhelmingly superior in terms of the dose distribution, the optimization time, and the reproducibility of an intended marginal dose, except for the higher total MU per fraction. Assuming the sufficient GTV coverage by the prescription dose, as in this study, excellent GTV dose conformity and the steepness of dose gradient outside the boundary in the VMA are clearly advantageous in terms of reducing the risk of adverse radiation effects. However, the irradiation must be initiated as early as possible after image acquisition to minimize the effects of possible tumor growth and/or displacement during the waiting period [28]. In the VMA, the dose attenuation margins outside the GTV were also appropriate in most cases; however, those were too steep in some small lesions [25]. The extremely steep dose falloff within the outer 2-3 mm of the GTV boundary may result in insufficient control of microscopic brain infiltration, especially in cases of small-cell lung cancer and malignant melanoma with a strong tendency to invade the surrounding brain tissue [25,29]. In such cases, the extremely steep dose attenuation margin needs to be adjusted [15,25].

In the two treatment plans in this study, although the settings were made as uniform as possible to ensure that the optimization conditions were optimal for both groups, the beamlet width and the target/avoidance margin of the intensity-modulated radiotherapy (IMRT) prescription parameters were different [15,16]. The impact of changing the beamlet size to 2 mm and the margin to very tight (0-1 mm) needs to be confirmed in VMA. In addition, it may be useful to add a negative margin option, e.g., -2 mm, to DCA to make the dose gradient at the target boundary steeper.

It should be mentioned that the dose prescription and dose distribution design applied in this study contain many highly unique elements compared to general SRS practices [1-3]. To the authors‘ knowledge, dose prescription to the GTV D_V-0.01 cc_ has not been reported elsewhere [24]. However, this is similar to the GTV coverage by an unspecified or vaguely defined “marginal dose” in SRS using the Leksell Gamma Knife (Elekta AB) and can be said to be a concrete quantification of the coverage value [24]. Furthermore, while a maximum dose constraint within the GTV is typically imposed in SRS planning, the dose distribution in this study was intentionally optimized without such a dose constraint to maximize the physical characteristics of the system used, resulting in extremely inhomogeneous and highly variable doses within the GTV [18]. There are still many radiation oncologists who prefer relatively homogeneous target doses, such as those achieved with conventional three-dimensional conformal radiotherapy and who are resistant to extremely inhomogeneous target doses with steep dose gradients. However, previous planning studies using various devices have demonstrated that allowing a high dose inside the target is more likely to result in dose reduction in the tissues surrounding the target and a higher consistency of the prescription IDS with the target boundary [20]. The steep dose increase inside the GTV boundary may overcome the inherent radioresistance associated with hypoxic regions within the GTV and lead to anti-tumor effects such as earlier and more extensive development of tumor necrosis [26]. However, it may also induce tumor bleeding and/or swelling, especially using a single high dose with a few fractions. In ≥5-fraction SRS for large lesions, the internal dose escalation often contributes to early tumor shrinkage during the irradiation period; however, consideration must be given to the need for adaptive planning for possible tumor shrinkage and displacement [30].

The optimization time was unexpectedly shorter for the VMA. It may be more difficult to create an ideal distribution with highly conformal radiation fields that follow the target shape. In a typical DCA dose distribution, the high dose areas tend to be concentrated in the cranial region within the TV; however, in VMA, a steep concentrically layered dose gradient inside the boundary can be achieved by including partial irradiation fields that are limited to the caudal region [26]. As expected, the total MU value was significantly higher in the VMA; however, the VMA mainly consisted of the stacking of partial irradiation fields to portions of the target configuration, which inevitably resulted in a decrease in efficiency.

DCA with the HDRS may be suitable for SRS of the resection cavity of BMs, which requires different dose prescriptions and dose distribution designs compared with unresected cases [1-3]. The boundary between the cavity and the brain tissue is frequently irregular and unclear, and the shape and position of the cavity can change depending on the number of days since the surgery. Unless partially resected cases, the target of tumor control is the microscopic brain infiltration area, and the resection cavity is mostly filled with liquid components mainly consisting of cerebrospinal fluid. The dose required for tumor bulk control is not required, and the significance of the internal dose escalation is low. For targets with respiratory motion, such as lung tumors, DCA may be more effective than VMA in ensuring the target dose [11-13].

Study limitations

In this study, the settings of arc rotation ranges, in addition to the beamlet widths and target/avoidance margins, were different between DCA and VMA. The arc arrangements used as templates in actual clinical practices were adopted for DCA and VMA; however, the same settings may make the difference between optimization and dose distribution clearer. This study also did not include comparisons of the number of control points or the actual beam-on times. Furthermore, this comparative study of DCA and VMA only covered treatment planning; therefore, it is necessary to determine the final superiority or inferiority and the clinical conditions suitable for each, after comparing the dose verification and the actual treatment outcomes.

## Conclusions

In SRS using the HDRS for unresected solitary BMs, compared with DCA, VMA showed significant superiority in terms of the quality and efficiency of treatment planning, although with a higher total MU per fraction. In particular, the VMA dose distribution was overwhelmingly superior regarding the GTV dose conformity, the steepness and the concentric lamellarity of dose gradients outside and inside the GTV boundary, and the appropriateness of the dose attenuation margin. VMA was, therefore, positioned as the top-priority technique for SRS using the HDRS for intact BMs. However, sufficient consideration must be given to the extremely steep dose attenuation margin and internal dose increase at the GTV boundary, depending on the case.
